# Resident satisfaction with the pediatric surgery training program

**DOI:** 10.1186/s12909-020-02309-9

**Published:** 2020-10-20

**Authors:** Tariq Altokhais, Mohammed Al Rajhi, Osama Bawazir, Gassan T. Almogbel, Abdullah I. Aljunaydil, Abdullah Alshehri

**Affiliations:** 1 Division of pediatric surgery, Department of surgery, College of Medicine and King Saud University Medical City, King Saud University, P.O. Box: 1145111, Riyadh, 4545 Saudi Arabia; 2 Division of pediatric surgery, Security Forces Hospital, Riyadh, Saudi Arabia; 3 Department of Surgery, Faculty of Medicine, Umm Al-Qura University, Makkah, Saudi Arabia; 4 College of Medicine and King Saud University Medical City, King Saud University, Riyadh, Saudi Arabia

**Keywords:** Pediatric, Surgery, Resident, Satisfaction

## Abstract

**Background:**

The pediatric surgery residency program is new in Saudi Arabia. As with any new program, residents experience a degree of fear and anxiety about their future in the program. The aim of this study is to examine residents’ satisfaction with the program.

**Methods:**

This study included an online survey examining residents’ satisfaction. It consisted of demographic, financial, personality, program-specific, and burnout assessment questions. All questions were multiple-choice items.

Descriptive statistical data are presented as frequency distributions and percentages. Cross-tabulations and chi-square tests were used at the bivariate level of analysis to compare subgroups and identify factors of satisfaction. Binary logistics regression was used at the multivariate level of analysis to compute the odds ratio of significant variables.

**Results:**

Thirty-one out of 32 residents responded to the survey. The multivariate logistic regression showed that current year of residency, current relationship status and personality statistically affected the satisfaction of residents. Senior residents, i.e., residents who had spent four years or more in the program, were 40 times more likely to be satisfied than were residents in their first year; residents who were married were more than eight times more likely to be satisfied than were residents who were single; and residents who were neutral or who agreed that they were very indecisive were 8% less likely to be satisfied than were those who reported being decisive. Gender was statistically significant, such that males were more satisfied than females were.

**Conclusions:**

Although the pediatric surgery residency program is new, this survey has shown that there is generally a high rate of satisfaction. Satisfaction was also observed more in senior residents. Further studies should be conducted in the future when residents graduate from the program.

## Background

Residency training programs are diverse in regard to many aspects, such as structure, workload, expectations, and experience. Some publications have examined residents’ satisfaction and well-being in particular programs [1–3]. The pediatric surgery residency program is a six-year structured program. The training is run according to a nation- wide curriculum approved by the Saudi Commission for Health Specialties (the only official governor training body in Saudi Arabia). The first residents entered the program in October 2016. The structure of the program is divided into three levels; core surgery level in the first and second years, junior pediatric surgery level in the third and fourth years, and senior pediatric surgery level in the fifth and sixth years. Currently, there are fourteen accredited training centers with 60 faculty distributed in Saudi Arabia. It is a joint program where residents rotate in different centers according to their level of training and according to the scope of service for each training center. Each center accepts a resident every other year. Annually, seven residents are accepted to enter the program. There were only two residents dropped out so far.

Different tools of assessment, formative and summative, are used based on the curriculum. These tools are logbook for surgical procedures, end of year written exam, structured oral exam, objective structured practical exam (OSPE), first part written exam, and final written and clinical exams.

The program is considered highly competitive in the Saudi Commission for Health Specialties matching system and is one of the top five programs since it started. More details of the program are available as a supplement and can be accessed at www.scfhs.org.sa.

The residents are relatively young which makes them vulnerable to burnout and with any new program, residents experience a degree of fear and anxiety about their future in the program [4]. The aim of this study is to examine residents’ satisfaction with the program and the degree of burnout. To our knowledge, this is the first study of this program.

## Methods

This study was approved by the Institutional Review Board. Project No. E-20-5007 and a written consent was obtained from the study participants. In April 2020, a voluntary online google documents survey was electronically distributed to all the residents in the training program using the list of registration at the Saudi Commission for Health Specialties for pediatric surgery program, Saudi Arabia. The online-based survey consisted of 15 demographic, financial, and personality questions, 20 program-specific questions, and 22 burnout assessment questions. All questions are multiple-choice items. The full survey is available as a supplement.

The data processing and analysis were performed using the Statistical Package for Social Sciences (SPSS) (Armonk, NY, USA, version 24). Descriptive statistical data are presented as frequency distributions and percentages. Cross-tabulations and chi-square tests were used at the bivariate level of analysis to compare subgroups and identify factors of satisfaction. Binary logistics regression was used at the multivariate level of analysis to compute the odds ratio of significant variables.

Satisfaction was measured by asking residents to select responses on a scale on which dissatisfaction is indicated if they chose “disagree” or “strongly disagree” and satisfaction if they chose “neutral”, “agree”, or “strongly agree”.

For burnout questions, a dichotomous variable representing the satisfaction of residents was generated using all 22 items on the scale provided by the survey. A reliability test using Cronbach’s alpha was conducted to measure the internal consistency of the items or questions. A Cronbach’s alpha of 0.70 (*n* = 22) showed that the items have an accepted measure of internal consistency and can be used to generate a construct. Some questions were reversed to ensure that the responses from the questions were directional. The questions were thereafter compiled together. Respondents who had scores below 60 on the scale were categorized as satisfied, while respondents with scores above 60 on the scale were categorized as not satisfied.

## Results

The program was composed of a total of 32 residents. Thirty-one (96.8%) responded to the survey. Twenty-eight residents were approximately equally distributed by postgraduate year (PGY), from PGY-1 to PGY-4. Only three residents were in PGY-5. Nineteen females and 12 males were aged between 25 and 30 years. Most of the residents were single (64.5%). Half of the residents took care of other people similarly to how they would take care of their parents. Of those who took care of others, 4 (26.7%) provided financial help, such that they sent money regularly to their parents; 3 (20%) took care of their parents’ social needs, such as accompanying them to their appointments or social events; and 8 (53.3%) took care of both the financial and social needs of their parents. Most of the residents believed that their monthly salary was sufficient for basic requirements.

The bivariate analysis to determine the relationship between selected characteristics of the residents and satisfaction showed that there was a significant association between current year of residency and the satisfaction of residents (X^2^(4, *N* = 31) = 9.83, *p* < .05). Residents who had spent 4 years or more in the program were satisfied more than were residents in their first year. Similarly, there was a significant association between gender and the satisfaction of residents (X^2^(1, *N* = 31) = 4.29, *p* < .05) and between current relationship status and the satisfaction (X^2^(1, *N* = 31) = 6.23, *p* < .05). The percentages of respondents who were satisfied or not did not differ significantly across all other variables (Table 1).

**Table 1:** Bivariate Analysis of Socio-Demographic variables on Satisfaction of Residents

VARIABLE	Satisfied (%)	Not Satisfied (%)	Total (%)	Chi-Square	***p***-value
**What is your current Residency year?**					
1	2	6	8	9.83	0.043
	25.00%	75.00%	100.00%		
2	3	3	6		
	50.00%	50.00%	100.00%		
3	2	5	7		
	28.60%	71.40%	100.00%		
4	6	1	7		
	85.70%	14.30%	100.00%		
5	3	0	3		
	100.00%	0.00%	100.00%		
**Gender**					
Male	9	3	12	4.29	0.038
	75.00%	25.00%	100.00%		
Female	7	12	19		
	36.80%	63.20%	100.00%		
**Current relationship status**					
Single	7	13	20	6.23	0.013
	35.00%	65.00%	100.00%		
Married	9	2	11		
	81.80%	18.20%	100.00%		
**If you are married, does your spouse also work in a healthcare field?**					
Yes	3	2	5	1.59	0.207
	60.00%	40.00%	100.00%		
No	8	1	9		
	88.90%	11.10%	100.00%		
**Number of kids**					
0	7	5	12	2.95	0.229
	58.30%	41.70%	100.00%		
1	4	0	4		
	100.00%	0.00%	100.00%		
2	1	0	1		
	100.00%	0.00%	100.00%		
**Do you take care of others; like parents?**					
Yes	10	4	14	0.76	0.383
	71.40%	28.60%	100.00%		
No	6	5	11		
	54.50%	45.50%	100.00%		
**Monthly salary (Saudi Riyal):**					
Less than 20,000	11	10	21	0.02	0.901
	52.40%	47.60%	100.00%		
20,000 - 30,000	5	5	10		
	50.00%	50.00%	100.00%		

The bivariate analysis to determine the relationship between personality and the satisfaction of residents showed that there was a significant association between people who are indecisive and satisfaction (X^2^(1, *N* = 31) = 7.89, *p* < .05). Satisfaction did not differ among those with other personality types (Table 2).

**Table 2:** Bivariate Analysis of Personality on Satisfaction of Residents

Variable	Satisfied (%)	Not Satisfied (%)	Total (%)	Chi Square	***p***-value
**I would describe myself as a perfectionist**					
Disagree	1	2	3	0.44	0.505
	33.30%	66.70%	100.00%		
Agree or Neutral	15	13	28		
	53.60%	46.40%	100.00%		
**I would describe myself as very empathetic**					
Disagree	1	2	3	0.44	0.505
	33.30%	66.70%	100.00%		
Agree or Neutral	15	13	28		
	53.60%	46.40%	100.00%		
**I would describe myself as very indecisive**					
Disagree	10	2	12	7.89	0.005
	83.30%	16.70%	100.00%		
Agree or Neutral	6	13	19		
	31.60%	68.40%	100.00%		
**I would describe myself as very idealistic**					
Disagree	3	1	4	1.01	0.316
	75.00%	25.00%	100.00%		
Agree or Neutral	13	14	27		
	48.10%	51.90%	100.00%		

**Fig. 1:**
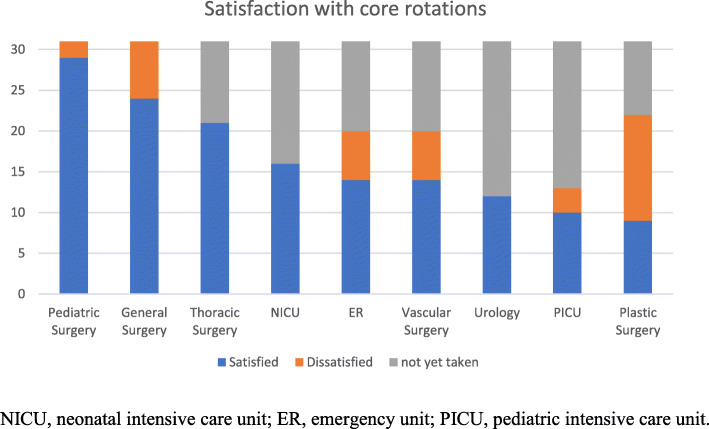
Satisfaction with core rotations. NICU, neonatal intensive care unit; ER, emergency unit; PICU, pediatric intensive care unit

The bivariate analysis to determine the relationship between faculty or program characteristics and the satisfaction of residents showed that satisfaction did not differ at the 95% confidence interval with respect to faculty or program characteristics (Table 3). The residents rotate in different core rotations. They were asked about their satisfaction in those rotations. All rotations were satisfactory except the plastic surgery rotation (Fig. 1).

**Table 3:** Bivariate Analysis of Faculty/Program Characteristics on Satisfaction of Residents

Variable	Satisfied (%)	Not Satisfied (%)	Total (%)	Chi Square	***p***-value
**Faculty and staff in my program care about my educational success.**					
Disagree	0	1	1	1.10	0.294
	0.00%	100.00%	100.00%		
Agree or Neutral	16	14	30		
	53.30%	46.70%	100.00%		
**My program uses resident feedback constructively.**					
Disagree	5	1	6	3.00	0.083
	83.30%	16.70%	100.00%		
Agree or Neutral	11	14	25		
	44.00%	56.00%	100.00%		
**I am able to have an adequate balance between work and personal life.**					
Disagree	4	6	10	0.80	0.372
	40.00%	60.00%	100.00%		
Agree or Neutral	12	9	21		
	57.10%	42.90%	100.00%		
**I often have a workload that results significant stress.**					
Disagree	2	0	2	2.00	0.157
	100.00%	0.00%	100.00%		
Agree or Neutral	14	15	29		
	48.30%	51.70%	100.00%		
**I would choose the same pediatric surgery residency program again if I had the chance.**					
Disagree	0	1	1	1.10	0.294
	0.00%	100.00%	100.00%		
Agree or Neutral	16	14	30		
	53.30%	46.70%	100.00%		
**I feel there is a lot of ambiguity and vagueness in the program.**					
Disagree	4	6	10	0.80	0.372
	40.00%	60.00%	100.00%		
Agree or Neutral	12	9	21		
	57.10%	42.90%	100.00%		
**I am afraid about the future after graduation from the program in terms of: I feel I will not have enough knowledge of pediatric surgery.**					
Disagree	11	7	18	1.55	0.213
	61.10%	38.90%	100.00%		
Agree or Neutral	5	8	13		
	38.50%	61.50%	100.00%		
**I am afraid about the future after graduation from the program in terms of: I feel I will not have enough surgical skills.**					
Disagree	4	6	10	0.80	0.372
	40.00%	60.00%	100.00%		
Agree or Neutral	12	9	21		
	57.10%	42.90%	100.00%		
**I am afraid about the future after graduation from the program in terms of: I feel I will not be accepted/employed in any respectful Hospital/institution.**					
Disagree	6	5	11	0.06	0.809
	54.50%	45.50%	100.00%		
Agree or Neutral	10	10	20		
	50.00%	50.00%	100.00%

For burnout, 22 questions were asked, and the results showed that 16 (51.6%) respondents were satisfied, while 15 (48.4%) respondents were not satisfied.

The multivariate logistic regression to determine the factors affecting the satisfaction of residents showed that their current year of residency, current relationship status, personality statistically, and gender affect their satisfaction. Senior residents, i.e., residents who had spent 4 years or more in the program, were 40 times more likely to be satisfied than were residents in their first year (O.R. = 40.160, *p* < .05); residents who were married were more than eight times more likely to be satisfied than were residents who were single (O.R. = 8.36, *p* < .05); and residents who were neutral or who considered themselves to be very indecisive were 8% less likely to be satisfied than were residents who reported being decisive (O.R. = 5.14, *p* < .05). Gender was statistically significant, such that males were more satisfied than females were (Table 4).

**Table 4:** Multivariate Logistics Regression; Satisfaction of Residents

Variable	*p-value*	Odds Ratio	95% Confidence Interval
**What is your current Residency year?**			
1	RC		
2	.341	3.00	0.312–28.841
3	.876	1.20	0.121–11.865
4	.033	18.00	1.267–255.744
5	.999	484^e5^	–
**Gender**			
Female	RC		
Male	.046	5.143	1.033–25.602
**Current Relationship Status**			
Single	RC		
Married	.020	8.357	1.400–49.883
**I would describe myself as very indecisive**			
Disagree	RC		
Agree or Neutral	.009	0.092	0.015–0.559

*RC* Reference category

## Discussion

The pediatric surgery training program is new in Saudi Arabia. With any new program, anxiety and many other concerns may arise regarding the program and the future of the gradates. We conducted this survey 4 years after the beginning of the program to examine and explore residents’ satisfaction with the program. Resident satisfaction is an important performance metric for surgery programs. Low satisfaction can lead to resident attrition [3]. Increased satisfaction potentially improves the performance, productivity, and retention of residents [3–7].

From the survey, there was a significant association between current year of residency and the satisfaction of residents, where satisfaction improved significantly when they became seniors. The more senior the resident, the more satisfied they were. Senior residents, i.e., residents who had spent 4 years or more in the program, were 40 times more likely to be satisfied than were residents in their first year. This finding could be explained by the fact that residents learned more as they progressed through the program, or may be due to the many rotations they have to go through during their more junior years, which may in turn affect their satisfaction.

There was a significant association between gender and the satisfaction of residents, and there was a statistically significant association between current relationship status and satisfaction. Single female residents were less satisfied with the program than were males or married residents.

Demographic and socioeconomic factors have been reported as reasons for dissatisfaction in the literature [8], but in this survey, these factors were not related to differences in satisfaction.

Most of the residents were satisfied with the program structure, faculty, and different rotations. All of the residents except one would choose the same pediatric surgery training program if they had the chance.

The survey contained 22 questions about burnout. Although most residents were satisfied with the program in general, half of them were dissatisfied when answering burnout questions. Burnout is experienced as emotional exhaustion or depersonalization [9, 10]. A recent study showed a burnout rate of 43.9% among physicians in the United States [11]. Burnout may lead to physical and emotional withdrawal, which may adversely affect patient care [11].

Training program satisfaction surveys may guide to implement interventions that improve satisfaction and reduce burnout [1]. Using residents’ feedback constructively and controlling the work load improve the program. The survey may help to address certain potential areas in the program that needs to be improved and lead to increase residents’ satisfaction. Although the curriculum for the program is regularly updated, findings from this survey maybe considered in the updates to reflect residents’ opinion and feedback.

This survey had some limitations. Although the response rate was 96.8%, the total number of residents was small. In addition, the survey might have produced different results if it had been distributed after a few years, when some residents had graduated from the program.

## Conclusion

Although the pediatric surgery residency program is new, this survey has shown that there is generally a high rate of satisfaction. Satisfaction was also observed more in senior residents. Further studies should be conducted in the future when residents graduate from the program.

## Data Availability

All data used in the study are available to be reviewed.

## Abbreviations

PGY: Postgraduate year
